# Polymorphisms Associated with Age at Onset in Patients with Moderate-to-Severe Plaque Psoriasis

**DOI:** 10.1155/2015/101879

**Published:** 2015-11-03

**Authors:** Rocío Prieto-Pérez, Guillermo Solano-López, Teresa Cabaleiro, Manuel Román, Dolores Ochoa, María Talegón, Ofelia Baniandrés, José Luis López-Estebaranz, Pablo de la Cueva, Esteban Daudén, Francisco Abad-Santos

**Affiliations:** ^1^Clinical Pharmacology Service, Hospital Universitario de la Princesa, Instituto Teófilo Hernando, Instituto de Investigación Sanitaria Princesa (IP), Universidad Autónoma de Madrid (UAM), 28006 Madrid, Spain; ^2^Dermatology Service, Hospital Universitario de la Princesa, Instituto de Investigación Sanitaria Princesa (IP), 28006 Madrid, Spain; ^3^Centro de Investigación Biomédica en Red de Enfermedades Hepáticas y Digestivas (CIBERehd), Instituto de Salud Carlos III, Madrid, Spain; ^4^Dermatology Service, Hospital Universitario Gregorio Marañón, 28007 Madrid, Spain; ^5^Dermatology Service, Hospital Universitario Fundación Alcorcón, 28922 Madrid, Spain; ^6^Dermatology Service, Hospital Universitario Infanta Leonor, 28031 Madrid, Spain

## Abstract

Psoriasis is a chronic skin disease in which genetics play a major role. Although many genome-wide association studies have been performed in psoriasis, knowledge of the age at onset remains limited. Therefore, we analyzed 173 single-nucleotide polymorphisms in genes associated with psoriasis and other autoimmune diseases in patients with moderate-to-severe plaque psoriasis type I (early-onset, <40 years) or type II (late-onset, ≥40 years) and healthy controls. Moreover, we performed a comparison between patients with type I psoriasis and patients with type II psoriasis. Our comparison of a stratified population with type I psoriasis (*n* = 155) and healthy controls (*N* = 197) is the first to reveal a relationship between the *CLMN*, *FBXL19*, *CCL4L*, *C17orf51*, *TYK2*, *IL13*, *SLC22A4*, *CDKAL1*, and *HLA-B/MICA* genes. When we compared type I psoriasis with type II psoriasis (*N* = 36), we found a significant association between age at onset and the genes *PSORS6*, *TNF-α*, *FCGR2A*, *TNFR1*, *CD226*, *HLA-C*, *TNFAIP3*, and *CCHCR1*. Moreover, we replicated the association between rs12191877 (*HLA-C*) and type I psoriasis and between type I and type II psoriasis. Our findings highlight the role of genetics in age of onset of psoriasis.

## 1. Introduction

Psoriasis is a chronic inflammatory skin disorder with a major genetic component. The prevalence of chronic plaque psoriasis is around 2% in the general population [1]. The many genetic studies performed in recent years showed that genes such as interleukin 23 receptor (*IL23R*) and* IL12B* and tumor necrosis factor alpha (*TNFα*) are closely associated with psoriasis and related diseases such as rheumatoid arthritis, psoriatic arthritis, and Crohn's disease [2]. Human leukocyte antigen C (HLA-C)^*∗*^0602 is the allele most closely associated with this disease [3].

The age at onset of psoriasis follows a bimodal distribution [4]: type I psoriasis appears before the age of 40 years (early-onset), with a peak at 16–22 years; type II psoriasis appears after the age of 40 years (late-onset), with a peak at 57–60 years [5]. Type I psoriasis has been associated with several single-nucleotide polymorphisms (SNPs) in genes associated with the immune response (Table 1). For example, HLA-C^*∗*^0602 is more strongly associated with type I psoriasis than with type II psoriasis [5]. Although several association studies have already been performed in psoriasis in both populations (type I or type II psoriasis patients), knowledge of age at onset remains limited and controversial (Table 1) [6]. Therefore, we performed a candidate gene study, where we evaluated genetic susceptibility to type I or type II psoriasis in patients with moderate-to-severe chronic plaque psoriasis. This approach may help us to identify SNPs previously associated with psoriasis or other autoimmune diseases [2] that are specific to type I or type II psoriasis. Furthermore, our genetic study could improve our understanding of psoriasis and of its etiology and pathogenesis.

## 2. Material and Methods

### 2.1. Experimental Design

We recruited 198 Caucasian patients with moderate-to-severe plaque type psoriasis (psoriasis area and severity index > 10) who attended the department of dermatology in four university hospitals in Madrid between 16/10/2007 and 17/12/2012. Five samples did not fulfill the quality criteria of the Human Genotyping Unit-CeGen (CEGEN, Spanish National Cancer Research Centre, Madrid, Spain), and 2 samples had insufficient volume. We also included 197 healthy volunteers (controls) recruited between 10/01/2011 and 14/12/2012 from the Clinical Pharmacology Service (Hospital Universitario de la Princesa, Madrid, Spain). All the volunteers were Caucasian and had no personal or family history of psoriasis (at least 2 generations).

The protocol fulfilled Spanish law on biomedical research and was approved by the Ethics Committee for Clinical Investigation of Hospital Universitario de la Princesa. All controls and patients gave their written informed consent to donate a sample for investigation. The samples are kept in the Clinical Pharmacology Service.

### 2.2. Selection of the Polymorphisms

We preselected 320 SNPs based on an extensive review of 449 articles describing the association between polymorphisms and psoriasis and response to biological drugs and psoriasis and related inflammatory diseases (rheumatoid arthritis, psoriatic arthritis, and Crohn's disease) [2]. We finally selected 192 SNPs based on minor allele frequency (≥0.05) and on the results of studies performed in Caucasians and psoriatic patients. Information on the 173 SNPs analyzed can be found in supplementary Table S1, which is published in [3].

### 2.3. Sample Processing

A 3-mL peripheral blood sample was extracted from each subject in EDTA tubes. DNA was obtained from samples using an automatic DNA extractor (MagNa Pure System, Roche Applied Science, USA) and its concentration was quantified in Nanodrop ND-1000 Spectrophotometer (Wilmington, USA). The extracted DNA was stored at −80°C in the Clinical Pharmacology Service until use.

### 2.4. Genotyping

A total of 196 samples from patients (2 samples of 198 cases had insufficient volume) and 197 samples from controls were sent to the Human Genotyping Unit-CeGen to genotype 192 SNPs. The analysis was performed using the Illumina Veracode genotyping platform. If fluorescence was low or the genotype clusters were undifferentiated, the SNPs were removed. In addition, if the call rate was less than 95% of the average of the 192 SNPs analyzed, the samples were removed. Since CEGEN quality criteria were not met in 19 SNPs and 5 patients, we finally analyzed 173 SNPs in 191 patients and 197 controls.

### 2.5. Statistical Analysis

The statistical analysis was performed to compare the following stratified populations: patients with type I psoriasis (*N* = 155) or type II psoriasis (*N* = 36) versus controls (*N* = 197) and patients with type I psoriasis versus cases with type II psoriasis. Hardy-Weinberg equilibrium was tested for all the SNPs analyzed using the SNPStats program [32]. Allele and genotype frequencies were also calculated using the SNPStats program. SNPs that were not in Hardy-Weinberg equilibrium in controls were removed from the subsequent analysis [33].

The univariate analysis was performed using R 3.0.2. (SNPassoc) [34]. We constructed various logistic regression models depending on the main types of inheritance (codominant, dominant, recessive, and additive). In the additive model, the presence of 2 mutant alleles confers double the risk of 1 mutant allele [33]. The results were adjusted for rs12191877 (SNP that is strongly associated with the HLA-C^*∗*^0602 allele and is highly prevalent in our population) [3, 35]. The optimal model was selected using the lower Akaike Information Criterion (AIC). Subsequently, SNPs with *p* < 0.1 in the univariate analysis (adjusted for rs12191877) were included in a multivariate logistic regression model to adjust for relevant confounding factors (SPSS 15.0). The results of the univariate analysis were adjusted for rs12191877, except when we compared patients with type I psoriasis and patients with type II psoriasis (the influence of rs12191877 was not very relevant). We expressed the results as the odds ratio (OR), 95% confidence interval, and *p* value.

## 3. Results

### 3.1. Study Population

The study population included 155 patients with moderate-to-severe chronic plaque type I psoriasis (92 men and 63 women), 36 patients with type II psoriasis (19 men and 17 women), and 197 controls (98 men and 99 women). The mean age was 46.01 ± 13.11 years in patients with type I psoriasis (45.72 ± 11.69 in men and 46.43 ± 15.04 in women), 67.72 ± 11.85 years in patients with type II psoriasis (65.95 ± 11.18 in men and 69.71 ± 12.59 in women), and 24.51 ± 4.29 years in the controls (25.07 ± 4.94 in men and 23.95 ± 3.46 in women). The mean age at onset of psoriasis was 23.31 ± 8.52 in patients with type I psoriasis and 52.58 ± 10.45 in patients with type II psoriasis. Analysis of the effect of sex on our results revealed no significant association.

### 3.2. Genotyping Results

A total of 192 SNPs were analyzed (see supplementary Table S1 published in [3]). However, only 173 SNPs fulfilled the quality criteria. One SNP was monomorphic (rs165161 in the* JUNB* gene) and was excluded from the statistical analysis. The genotyping success rate was 89.82%, and the reproducibility rate was 100%.

All the minor allele frequencies were in Hardy-Weinberg equilibrium except 9 SNPs in the controls and 12 SNPs in the patients (see supplementary Table S1 published in [3]). The 9 SNPs which were not in Hardy-Weinberg equilibrium in controls were removed from the statistical analysis [33].

### 3.3. Association with Type I or Type II Psoriasis

Our findings showed an association between type I psoriasis and 10 SNPs (*N* = 155 versus *N* = 197 controls): rs1634517 (*CCL4L*), rs1975974 (*C17orf51*), rs12720356 (*TYK2*), rs1800925 (*IL13*), and rs6908425 (*CDKAL1*) decreased the risk of psoriasis 2.94-fold, 2.08-fold, 10-fold, 100-fold, and 2.44-fold, respectively; and rs2282276 (*CLMN*), rs10782001 (*FBXL19*), rs3792876 (*SLC22A4*), rs12191877 (*HLA-C*), and rs13437088 (*HLA-B/MICA*) increased the risk of psoriasis 3.90-fold, 2.10-fold, 3.75-fold, 30.54-fold, and 2.52-fold, respectively (Table 2). However, comparison of 36 patients with type II psoriasis and 197 controls revealed no significant association (results not shown).

Four SNPs were associated with significant decreases in the risk of type I psoriasis (*N* = 155) compared with type II psoriasis (*N* = 36), namely, rs191190 (*TNFR1*; 126.08-fold), rs361525 (*TNF-α*; 190.76-fold), and rs10499194 and rs6920220 (*TNFAIP3*; 155.02-fold and 19.14-fold, resp.). We also found 5 SNPs that were associated with a significant increase in the risk of type I psoriasis, namely, rs1801274 (*FCGR2A*; 5.26-fold), rs763361 (*CD226*; 33.3-fold), rs12459358 (*PSORS6*; 11.11-fold), rs12191877 (*HLA-*C; 12.5-fold), and rs1576 (*CCHCR1*; 166.66-fold) (Table 3).

## 4. Discussion

About 75% of patients with chronic plaque psoriasis have type I psoriasis before age 40 [4], whereas a lower number of patients develop psoriasis at around 50–60 years [11]. Our results are consistent with these findings, since 79.06% of our patients developed psoriasis before the age of 40.

When we compared patients with type I psoriasis and controls, we found 10 significant SNPs in* CLMN*,* FBXL19*,* CCL4L*,* C17orf51*,* TYK2*,* IL13*,* SLC22A4*,* CDKAL1*,* HLA-C*, and* HLA-B/MICA*.

The HLA-C^*∗*^0602 allele is a risk factor for psoriasis [35] and has been associated with both type I [6–9] and type II psoriasis [10]. In one study, 85.3% of patients with type I psoriasis had this allele [5], whereas only 14.7% of patients with type II psoriasis were carriers [5]. Other authors found an association between rs10484554 (*HLA-C*) and type I psoriasis compared with type II psoriasis (OR = 3.24 in type I) [12]. rs10484554 has also been associated with type II psoriasis [10]. In a recent GWAS, the* HLA-C* gene was associated with type I psoriasis (*p* = 2.97*E* − 18 for rs1265181, *p* = 2.58*E* − 15 for rs12191877, *p* = 1.84*E* − 15 for rs4406273, and *p* = 1.10*E* − 07 for rs2395029), but not with type II psoriasis after application of the Bonferroni correction [11]. In addition, our results showed significant differences in rs12191877 (*HLA-C*) in patients with type I psoriasis (*p* = 2.50*E* − 19). However, we did not find this association in patients with type II psoriasis, probably owing to the small sample size in this group (*N* = 36).

Munir et al. found an association between rs1295685 in the* IL13* gene and type I psoriasis (*p* = 2.47*E* − 03) [11]. Our results showed an association between another SNP in* IL13* (rs1800925) and type I psoriasis (*p* = 0.034). In addition, Munir et al. did not obtain significant results when they compared controls with type II psoriasis or type I psoriasis with type II psoriasis [11]. Both SNPs in* IL13* have been associated with predisposition to psoriasis [36, 37].

Our comparison of patients with type I psoriasis and controls is the first to obtain significant results for a series of SNPs in type I psoriasis, although the SNPs have already been associated with the risk of psoriasis. rs10782001 in* FBXL19* [38], rs1975974 in* C17orf51* [38], rs12720356 in* TYK2* [3, 39], rs3792876 in* SLC22A4* [3], rs6908425 in* CDKAL1* [40], and rs13437088 in* HLA-B/MICA* [35] have previously been associated with psoriasis, but not with type I psoriasis.

Furthermore, SNPs in* CLMN* (rs2282276) and* CCL4L* (rs1634517) have not been associated with psoriasis or age at onset.

We found no significant differences between patients with type II psoriasis and controls owing to the small sample size (*N* = 36).

Comparison between patients with type I psoriasis and patients with type II psoriasis revealed significant associations for the following genes:* FCGR2A*,* TNFR1*,* CD226*,* PSORS6*,* TNFAIP3*,* HLA-C*,* TNF-α*, and* CCHCR1*.

Polymorphisms in* CCHCR1* (−386 and −404, CCHCR1^*∗*^WW allele) have been associated with type I psoriasis [9, 31]. We found significant differences between rs1576 in* CCHCR1* and age at onset. In a study comparing controls (54.8%) and patients with psoriasis type II (66.0%), Allen et al. showed a significant increase in the number of patients carrying rs1576 [41]. This SNP has been associated with psoriasis elsewhere [42].

Douroudis et al. analyzed rs763361 in* CD226* in patients with early-onset psoriasis and patients with late-onset psoriasis, although they found no associations [43]. We performed the same analyses and found significant differences between the groups. In addition, rs763361 in* CD226* has been associated with severity of psoriasis [43].

rs12459358 in* PSORS6* has been associated with type I psoriasis (G risk allele, OR = 1.47 and *p* = 0.005) [19]. In contrast, our data showed an association between the T allele and type I psoriasis (OR = 11.11; *p* = 0.026).

rs361525 (−238) in the* TNFα* gene has been associated with susceptibility to psoriasis [44], and the A allele was more frequent in male patients with type I psoriasis (*p* = 2*E* − 07) [15, 22]. We found significant results in rs361525 (*TNF-α*) when we compared patients with type I psoriasis and patients with type II psoriasis, although we found no gender differences. Other authors confirmed our association with type I psoriasis in Caucasian [20, 23] and Mongolian patients [24]. A meta-analysis showed an association between rs361525 and type I psoriasis [21]. Baran et al. found no significant differences between rs1800629 in the −308 promoter (*TNFα*) and type I or type II psoriasis [45].

Likewise, rs12191877 in* HLA-C* has been associated with increased risk of psoriasis [35]. Munir et al. [11] compared patients with type I psoriasis and patients with type II psoriasis and obtained significant results for rs1265181, rs4406273, and rs12191877 in* HLA-C*. We replicated these results in rs12191877 (T allele risk; *p* = 0.045).

rs191190 in* TNFR1* [46] and rs10499194 in* TNFAIP3* [3] have been associated with psoriasis, but not with age of onset. Moreover, rs1801274 in* FCGR2A* and rs6920220 in* TNFAIP3* have not been studied in patients with psoriasis according to age of onset. Given the small sample size in the group with type II psoriasis in our study, our results should be interpreted with caution.

Our results highlight the role of the immune system in psoriasis and enhance our understanding of pathogenic mechanisms. Such knowledge can help to optimize treatment.

Our study is subject to a series of limitations. First, mean age varied between the cases and the controls. Second, the sample size was limited by the number of study patients treated in the dermatology department, thus making it difficult to detect SNPs with a low probability of causing psoriasis. Third, since the SNPs were selected based on a literature review, several major SNPs may not yet have been investigated.

In conclusion, our study confirmed an association between rs12191877 (*HLA-C*) and type I psoriasis and between type I and type II psoriasis patients. Ours is the first study to show an association between* CLMN*,* FBXL19*,* CCL4L*,* C17orf51*,* TYK2*,* IL13*,* SLC22A4*,* CDKAL1*, and* HLA-B/MICA* and type I psoriasis. Moreover,* CLMN* and* CCL4L* have not been previously described in psoriasis. In addition,* PSORS6* and* TNFα* have been described as more prevalent genes in type I psoriasis and we showed a significant association when we compared type I psoriasis and type II psoriasis. Ours is the first study to identify an association between* FCGR2A*,* TNFR1*,* CD226*,* TNFAIP3*, and* CCHCR1* and age at onset of psoriasis. Our results suggest that genetics could play a role in age at onset. However, further studies are needed to confirm our findings.

## Figures and Tables

**Table 1:** SNPs associated with type I (early-onset) and type II (late-onset) psoriasis: an update.

SNP	Gene	Function^†^	Association with			References
			Ps type I	Ps type II	Ps type I versus type II	
—	HLA-C^*∗*^0602		X	X		[6–10]
—	HLA-C^*∗*^12:02			X		[8]
rs1265181	*HLA-C*	Encodes a class I molecule which plays a central role in the immune system by presenting peptides derived from endoplasmic reticulum lumen	X		X	[11]
**rs12191877**			X^*∗*^		X^*∗*^	[7, 11]
rs4406273			X		X	[11]
rs2395029			X			[11]
rs10484554			X	X	X	[7, 10, 12]
rs13191099				X		[4]
rs10876882	*HLA-A*			X		[4]

rs33980500	*TRAF3IP2*	Encodes a protein involved in regulating responses to cytokines by members of the Rel/NF-kappa-B transcription factor family	X			[11]
rs71562288				X		[4]

rs2233278	*TNIP1*	Encodes A20-binding protein which plays a role in autoimmunity and tissue homeostasis through the regulation of nuclear factor kappa-B activation			X	[11]
rs17728338	*TNIP1*				X	[11]

rs1295685	*IL13*	Encodes a cytokine involved in several stages of B cell maturation and differentiation	X			[11]

rs17716942	*IFIH1*	Encodes an Asp-Glu-Ala-Asp box protein (putative RNA helicases)	X			[7]
rs1990760				X		[4]

rs27524	*ERAP1*	Encodes an aminopeptidase involved in trimming HLA class I-binding precursors	X			[6]^#^

rs11209026	*IL23R*	Encodes a subunit of the receptor for IL23A/IL23	X			[6]^#^
rs72676067				X		[4]

rs10876882	*IL23A*	Encodes a subunit of IL23 involved in immune responses		X		[4]

—	*LCE3B/LCE3C-del*	Encodes precursors of the cornified envelope of the stratum corneum	X			[6, 13]^#^

rs2546890	*IL12B*	Encodes a subunit of IL12 that acts on T and natural killer cells	X	X		[4, 14]

rs60813083	*RNF114*	Encodes a protein that may play a role in spermatogenesis		X		[4]

rs887998	*IL1R1*	Encodes a receptor for IL1 involved in inflammatory responses		X		[4]

rs16944	*IL1B*	Encodes a cytokine produced by activated macrophages and is involved in immune responses, cell proliferation, differentiation, and apoptosis		X		[4, 15]
rs2853550				X		[4]

rs26653	*ERAP1*	Encodes an aminopeptidase involved in trimming HLA class I-binding precursors so that they can be presented on the MHC class I molecule	X			[10]
rs30187			X			[10]

rs2227473	*IL22*	Encodes an interleukin 22 that contributes to the inflammatory response	X			[16]
rs2227483			X			[16]
INDEL rs35774195/rs10784699			X			[16]

rs6822844	*IL2/IL21*	Encode cytokines that are important in the innate and adaptive immune responses by inducing differentiation, proliferation, and activity of multiple target cells including macrophages, natural killer cells, B cells, and cytotoxic T cells	X			[17]
rs2069778			X			[17]

rs6311	*HTR2A*	Encodes a receptor for neurotransmitter serotonin		X		[18]

**rs12459358**	*PSORS6*	Encodes genetic locus associated with susceptibility to psoriasis	X		*∗*	[19]

rs1800629	*TNF-α*	Encodes a cytokine secreted by macrophages and involved in the regulation of cell proliferation, differentiation, and apoptosis, as well as in lipid metabolism and coagulation	X			[20, 21]
**rs361525**			X		*∗*	[15, 20–24]

rs3733197	*BANK1*	Encodes a protein involved in B cell receptor-induced calcium mobilization from intracellular stores	X			[25]

rs755622	*MIF*	Encodes a lymphokine involved in cell-mediated immunity, immunoregulation, and inflammation		X	X	[26]

rs6693899	*IL10*	Encodes a cytokine produced by monocytes and lymphocytes and involved in immunoregulation and inflammation		X	X	[27]
rs1800896				X		[28]

rs4341	*ACE*	Encodes an enzyme involved in catalyzing the conversion of angiotensin I into a physiologically active peptide angiotensin II	X			[29]

SNPs at positions -1540, -1512, -1451, -460, and -152	*VEGFA*	Encodes a protein involved in angiogenesis, vasculogenesis, endothelial cell growth, promotion of cell migration, and inhibition of apoptosis		X		[30]

SNPs at positions -386 and -404	*CCHCR1*	Encodes a protein that may be a regulator of keratinocyte proliferation or differentiation	X			[31]
—	CCHCR1^*∗*^WW allele		X			[9]

SNP: single-nucleotide polymorphism; Ps: psoriasis; ^†^Information available at NCBI (http://www.ncbi.nlm.nih.gov/gene) or GeneCards (http://www.genecards.org/); ^#^Study performed in pediatric-onset psoriasis (patients <18 years); ^*∗*^Association found in our study.

**Table 2:** Results of univariate linear regression analysis (unadjusted and adjusted for rs12191877 in *HLA-C*) and multivariate linear regression analysis (155 patients with type I psoriasis versus 197 controls). In the multivariate analysis, we included the SNPs with *p* < 0.1 in the univariate analysis adjusted for *HLA-C*. Only polymorphisms that were significant in the multivariate analysis are shown.

SNP	Gene	Model	Risk genotype	Univariate unadjusted. Type I patients versus controls		Univariate adjusted for HLA-C		Multivariate	
				OR (95% CI)	*p* value	OR (95% CI)	*p* value	OR (95% CI)	*p* value
rs2282276	*CLMN*	A	CC/CT	1.74 (0.96–3.15)	0.066	1.95 (1.04–3.65)	0.037	3.90 (1.13–13.38)	0.031
rs10782001	*FBXL19*	A	GG/AG	1.58 (1.13–2.21)	0.007	1.59 (1.09–2.32)	0.016	2.10 (1.05–4.17)	0.035
rs1634517	*CCL4L*	D	AA/AC	0.89 (0.58–1.36)	0.590	0.64 (0.39–1.05)	0.073	0.34 (0.14–0.84)	0.019
rs1975974	*C17orf51*	A	GG/AG	0.80 (0.57–1.14)	0.220	0.66 (0.44–0.99)	0.040	0.48 (0.23–0.99)	0.048
rs12720356	*TYK2*	A	GG/GT	0.42 (0.21–0.81)	0.019	0.27 (0.13–0.58)	0.0003	0.10 (0.03–0.39)	0.001
rs1800925	*IL13*	R	TT	0.18 (0.02–1.45)	0.051	0.17 (0.02–1.49)	0.061	0.01 (0.00–0.73)	0.034
rs3792876	*SLC22A4*	A	TT/CT	1.57 (0.89–2.76)	0.110	1.87 (0.98–3.55)	0.057	3.75 (1.19–11.83)	0.024
rs6908425	*CDKAL1*	A	TT/CT	0.67 (0.47–0.97)	0.029	0.58 (0.39–0.89)	0.01	0.41 (0.20–0.85)	0.017
rs12191877	*HLA-C*	A	TT/CT	5.92 (3.83–9.15)	2.50*E* − 19	—	—	30.54 (10.62–87.85)	0.000
rs13437088	*HLA-B/MICA*	D	TT/CT	2.17 (1.42–3.34)	3.00*E* − 04	1.93 (1.19–3.13)	0.007	2.52 (1.01–6.31)	0.048

CLMN: calponin-like transmembrane gene; FBXL19: F-box and leucine-rich repeat protein 19; CCL4L: chemokine (C-C motif) ligand 4-like; C17orf51: chromosome 17 open reading frame 51; TYK2: nonreceptor tyrosine-protein kinase; IL13: interleukin 13; SLC22A4: solute carrier family 22 member 4; CDKAL1: cyclin-dependent kinase 5 regulatory subunit associated protein 1-like 1; HLA: major histocompatibility complex; MICA: major histocompatibility complex class I polypeptide-related sequence A; SNPs: single-nucleotide polymorphisms; OR: odds ratio of presenting type 1 psoriasis; CI: confidence interval; A: additive; R: recessive; D: dominant; —: no data.

**Table 3:** Results of univariate and multivariate linear regression analyses (155 patients with psoriasis type I versus 36 cases with psoriasis type II). SNPs with *p* < 0.1 in the univariate analysis were included in the multivariate analysis. Only polymorphisms that were significant in the multivariate analysis are shown.

SNP	Gene	Model	Risk genotype	Univariate Ps patients type I versus type II		Multivariate	
				OR (95% CI)	*p* value	OR (95% CI)	*p* value
rs1801274	*FCGR2A*	A	CC/CT	1.96 (1.12–3.45)	0.016	5.26 (1.11–25)	0.037
rs191190	*TNFR1*	D	CC/CT	0.43 (0.17–1.11)	0.065	0.01 (1.44*E* − 04–0.44)	0.018
rs763361	*CD226*	D	TT/CT	2.08 (0.99–4.35)	0.056	33.33 (1.11–1000)	0.043
rs12459358	*PSORS6*	A	TT/CT	2.44 (1.32–4.55)	0.002	11.11 (1.32–100)	0.026
rs10499194	*TNFAIP3*	D	TT/CT	0.38 (0.17–0.90)	0.02	0.01 (6.77*E* − 05–0.61)	0.030
rs12191877	*HLA-C*	A	TT/CT	2.33 (1.23–4.35)	0.006	12.50 (1.06–100)	0.045
rs6920220	*TNFAIP3*	A	AA/AG	0.55 (0.30–1.03)	0.068	0.05 (0.003–0.90)	0.042
rs361525	*TNF-α*	C	AG	2.17 (0.62–7.69)	0.087	0.01 (5.48*E* − 05–0.50)	0.024
rs1576	*CCHCR1*	D	GG/GC	2.56 (1.22–5.26)	0.012	166.67 (2.32–1000)	0.019

FCGR2A: Fc fragment of IgG low affinity IIa receptor; TNFR1: tumor necrosis factor receptor 1; CD226: CD226 antigen; PSORS6: psoriasis susceptibility 6; TNFAIP3: tumor necrosis factor alpha-induced protein 3; HLA-C: major histocompatibility complex; TNFAIP3: tumor necrosis factor alpha-induced protein 3; TNF-*α*: tumor necrosis factor alpha; CCHCR1: coiled-coil alpha-helical rod protein 1; SNPs: single-nucleotide polymorphisms; OR: odds ratio of presenting type I psoriasis; CI: confidence interval; A: additive; D: dominant; C: codominant.
